# Aging and HIV: Recent Findings in Contributing Factors

**DOI:** 10.1155/arat/8814760

**Published:** 2025-04-11

**Authors:** Flora Ramona Sigit Prakoeswa, Faradiba Maharani, Rochmadina Suci Bestari, Riandini Aisyah, Burhannudin Ichsan, Dodik Nursanto, Rizki Listiansyah, Muhammad Rizqy Noer Tuanaya

**Affiliations:** ^1^Department of Dermatovenereology, Faculty of Medicine, Universitas Muhammadiyah Surakarta, Surakarta, Central Java, Indonesia; ^2^Department of Dermatovenereology, PKU Muhammadiyah Hospital, Surakarta, Indonesia; ^3^Department of Dermatology, Faculty of Medicine, Universitas Sebelas Maret, Surakarta, Central Java, Indonesia; ^4^Department of Parasitology, Faculty of Medicine, Universitas Muhammadiyah Surakarta, Surakarta, Central Java, Indonesia; ^5^Department of Molecular Biology, Faculty of Medicine, Universitas Muhammadiyah Surakarta, Surakarta, Central Java, Indonesia; ^6^Department of Public Health, Faculty of Medicine, Universitas Muhammadiyah Surakarta, Surakarta, Central Java, Indonesia; ^7^Department of Anatomy and Embryology, Faculty of Medicine, Universitas Muhammadiyah Surakarta, Surakarta, Central Java, Indonesia; ^8^Department of Medical Education, Faculty of Medicine, Universitas Muhammadiyah Surakarta, Surakarta, Central Java, Indonesia

**Keywords:** aging, factors, HIV

## Abstract

**Background:** Aging among people living with HIV (PLWH) presents multifaceted challenges influenced by antiretroviral therapy (ART), chronic inflammation, viral coinfections, stigma, multimorbidity, and immunosuppression.

**Methods:** This review synthesizes recent research findings to outline factors contributing to aging with HIV. A comprehensive literature search was done using electronic databases including PubMed, Web of Science, Google Scholar, and CINAHL with keywords “HIV,” “Aging,” “Elderly,” “Geriatrics,” “Older Adults,” “HIV Infections,” and “HIV/AIDS.

**Results:** Addressing age-related comorbidities, cognitive impairment, and non-AIDS events is imperative as older PLWH face increased morbidity and mortality rates compounded by coinfections such as HCV, HPV, TB, HSV, and bacterial infections. While ART is vital for viral suppression, it introduces challenges such as mitochondrial toxicity, metabolic disorders, and decreased CD4 cell counts, accelerating the aging process. Lifestyle factors, including smoking, substance abuse, malnutrition, sedentary behavior, and mental health conditions, further exacerbate aging in PLWH.

**Conclusions:** This study emphasizes the necessity of holistic approaches to meet the unique healthcare needs of older PLWH, with insights into immunosenescence, coinfections, disease progression, ART exposure, and lifestyle factors. Understanding these dynamics is crucial for improving health outcomes and quality of life in aging PLWH.

## 1. Introduction

Human immunodeficiency virus (HIV) has been extensively studied over the years, leading to more effective therapies. The success of antiretroviral therapy (ART) has been demonstrated by reduced mortality rates and the growing population of people living with HIV (PLWH) with increased life expectancy [1, 2]. In 2020, UNAIDS estimated that 8.1 million people aged 50 years or older living with HIV globally, constituting approximately 20% of the total PLWH population [3]. Projections from the Dutch ATHENA cohort estimate this number will rise to 73% by 2030, highlighting the growing need to address the aging process among PLWH [4].

Aging is characterized not only by chronological age but also by the deterioration of functional capacity and increased risks of disability, morbidity, and mortality [5]. The aging process in PLWH is influenced by various factors, including ART usage, chronic inflammations, viral coinfections, stigma, multimorbidity, and immunosuppression [4–6]. Early studies have identified several biomarkers to measure aging in PLWH, such as CD14+, CD163+, CRP, IL-6, D-dimer, and leukocyte telomere length [4, 7, 8]. These biomarkers These biomarkers provide insight into chronic systemic immune activation and inflammation.

This demographic shift necessitates careful understanding to provide better healthcare approaches for older PLWH. This group faces several challenges, including age-related comorbidities, polypharmacy, cognitive impairment, neurocognitive disorders, and non-AIDS events (NAEs) such as hepatic and kidney diseases [4, 9, 10]. Moreover, accelerated aging compared with the general population further compound these challenges, leading to immune dysfunction, vulnerability to infections, and poor vaccine responses [9]. Thus, estimated PLWH in 2030 will have three or more comorbidities [11]. According to the concerns, this review aims to synthesize recent research findings to provide a comprehensive overview of the factors contributing to aging with HIV.

## 2. Methods

A comprehensive literature search using electronic databases, including PubMed, Web of Science, Google Scholar, and CINAHL. The search strategy targeted studies published between 2013 and 2023, focusing on the intersection of aging and HIV. Keywords and Medical Subject Headings (MeSH) terms used in the search included “HIV,” “Aging,” “Elderly,” “Geriatrics,” “Older Adults,” “HIV Infections,” and “HIV/AIDS,” using Boolean operators (AND and OR) to refine search results.

Data synthesis involved a narrative summary of key findings extracted from the studies. Themes and patterns across studies were identified and synthesized to provide insights into the factors contributed of aging on individuals living with HIV/AIDS, including immunological aspects, coinfections, and other related factors.

## 3. Immunosenescence and Inflammation

Aging is associated with changes in the immune system, a process known as immunosenescence. This involves a progressive decline in immune function and dysregulation of immune responses. Key changes include thymic involution that results in a reduced output of new T cells leading to a decline in the diversity and functionality of the T cell repertoire, CD8^+^ T cells dysfunction, B cells alteration that results in reduced antibody responses and impaired immune memory, chronic low grade inflammation (“inflammaging”) characterized by the increased level of proinflammatory cytokines and increased activation of immune cells, and dysregulation of immune checkpoints, such as programmed cell death protein 1 (PD-1) and cytotoxic T-lymphocyte-associated protein 4 (CTLA-4), resulting in impaired immune regulation [12, 13]. These changes results in decreased immune responses, reduced vaccine efficacy, and increased susceptibility to infections and age-related diseases, especially among older PLWH.

Immunosenescence in older PLWH differs from the normal population. HIV infection induces chronic immune activation, persistent viral replication, and chronic antigenic stimulation, all of which exacerbate immune dysfunction and accelerate aging [9]. Older PLWH also exhibit an increased prevalence of noncommunicable diseases (NCDs) such as cardiovascular diseases and diabetes, further driven by persistent immune activation and chronic inflammation [2]. Chronic inflammation in HIV is fueled by mechanisms such as microbial translocation and dysregulated immune responses [2]. Increased expression of activation markers on immune cells, such as CD38 and HLA-DR driven by the continuous viral replication and the presence of viral antigens, leads to the persistent immune activation [14–17]. In addition, chronic inflammation in older PLWH has profound effects on basic laboratory parameters including the neutrophil-to-lymphocyte ratio (NLR) and systemic inflammatory index (SII) and offer cost-effective tools for monitoring systemic health. Elevated NLR is linked to increased cardiovascular risks, while platelet counts and platelet crit (PCT) offer further insights into inflammatory burden and treatment response [18].

Furthermore, the microbiome conditions in PLWH also differ significantly from HIV-negative individuals. Dysbiosis, characterized by imbalance in the composition and diversity of the microbiome leads to chronic inflammation. Studies have shown alterations in the gut microbiome of PLWH, including a decrease in beneficial bacteria (e.g., *Bacteroidetes*) and an increase in harmful bacteria (e.g., *Proteobacteria*) [19]. Increased gut permeability in PLWH allows microbial products like lipopolysaccharides (LPSs) to enter the bloodstream, activating monocytes and macrophages, leading to the production of proinflammatory cytokines [5]. The translocation of microbial products from the gut contributes to systemic inflammation in HIV. HIV infection affects the immune system, including the gut-associated lymphoid tissue (GALT), which plays a crucial role in maintaining a healthy microbiome. The loss of CD4+ T cells in PLWH can disrupt the balance of the immune response in the gut, leading to alterations in the microbiome, particularly an increase in pathogenic bacteria such as (e.g., *Proteobacteria*) and a decrease in beneficial bacteria (e.g., *Lactobacillales*) [20, 21]. In addition, the dysregulation of other immune cells, such as B cells and natural killer cells, can further impact the microbiome composition and function [22, 23].

Dysregulation of the immune response as a result of HIV infection affects the balance and regulation of the immune response. The virus targets and depletes CD4+ T cells, which play a crucial role in coordinating immune responses. The loss of CD4+ T cells impairs the immune system's ability to control infections, leading to chronic immune activation marked by persistent viral antigens, hyperinflammation, elevated proinflammatory cytokines (TNF-α and IL-6), and increased susceptibility to infections, malignancies, and non-AIDS-related comorbidities such as cardiovascular and metabolic disorders [19, 23]. Moreover, HIV infection dysregulates immune cell function by expanding atypical memory B cells, which elevate immune activation and inflammation, while also promoting chronic activation that exhausts NK cells through the upregulation of inhibitory receptors [24, 25]. Chronic inflammation is associated with increased risk of non-AIDS-related comorbidities, such as cardiovascular disease, neurocognitive impairment, and metabolic disorders. Managing chronic inflammation is an important aspect of HIV care to improve long-term health outcomes for individuals living with HIV.

## 4. Coinfections

Coinfections pose additional risks for older PLWH, as their immune systems are often compromised by the combined effects of HIV infection and aging. These coinfections, including hepatitis C virus (HCV), human papillomavirus (HPV), tuberculosis (TB), herpes simplex virus (HSV), and bacterial coinfections, significantly impact health outcomes and require careful management [26].

### 4.1. HCV Coinfection

HCV coinfection is particularly common among older PLWH, especially those with a history of injection drug use before harm reduction strategies were widely implemented. HCV accelerates liver disease progression in PLWH, leading to advanced fibrosis, cirrhosis, hepatocellular carcinoma (HCC), and other liver-related complications. It also hampers immune recovery by impairing CD4+ T cell restoration, even in individuals receiving ART. The combined effects of HCV and HIV exacerbate chronic inflammation, immune activation, and liver damage, leading to higher morbidity and mortality rates compared to individuals with either infection alone [27, 28].

### 4.2. HPV Coinfection

HPV coinfection is another prevalent issue among PLWH. Immunosuppression caused by HIV increases the likelihood of persistent HPV infection and the progression to high-grade squamous intraepithelial lesions (HSILs) or HPV-related cancers [29, 30]. These include cervical, anal, and oropharyngeal cancers, which are particularly concerning in older PLWH due to their decreased ability to mount effective immune responses. Early detection and treatment of HPV-related conditions are essential to mitigate these risks.

### 4.3. HSV Coinfection

HSV coinfection is characterized by recurrent oral and genital ulcers, which can be more severe and frequent in older PLWH. HSV can significantly reduce the quality of life by causing physical discomfort and emotional distress [31]. In addition, the chronic inflammation associated with HSV infection further complicates the health of PLWH, particularly as they age.

### 4.4. Bacterial Infections

Older PLWH are at increased risk for bacterial infections, such as pneumonia, urinary tract infections, and skin and soft tissue infections [26]. These infections often lead to more severe illness and complications, reflecting the cumulative impact of aging, immune dysfunction, and HIV-related comorbidities. Timely identification and management of bacterial infections are critical to preventing further health deterioration in this vulnerable population.

### 4.5. TB Coinfections and HIV

TB remains one of the most significant coinfections among PLWH and is a leading cause of mortality in this group. Older adults are particularly susceptible due to age-related immune decline and comorbidities. TB coinfection can exacerbate systemic inflammation and immune exhaustion, leading to poor treatment outcomes and increased mortality [32]. The 2013 Botswana AIDS Impact Survey showed that older adults (50–64 years) with TB have a low level of education, with a high prevalence of HIV/TB coinfection (21.9%) and a high HIV prevalence (44% for age 50–54, 40.6% for age 55–59, and 68.4% for age 60–64 years). In addition to low education levels, factors such as lack of awareness of TB or HIV status, delayed testing for TB, and negative social attitudes can contribute to TB/HIV coinfection in older adults [33].

TB coinfection exacerbates systemic inflammation, triggering immune activation and contributing to immune exhaustion, which impairs immune function with elevated markers such as CD38 and HLA-DR on CD8 cells, while older PLWH are particularly vulnerable due to compromised lung epithelial integrity and greater susceptibility to lung damage [15, 34]. Elevated inflammatory biomarkers like IL-6 are linked to both active TB and cognitive impairment, and chronic inflammation from TB–HIV coinfection further increases the risk of cardiovascular disease, complicating treatment outcomes in older patients [35, 36].

Earlier studies showed that individuals with TB/HIV coinfection had higher mortality rates compared to those without HIV infection, especially when HIV was not treated. Factors associated with higher mortality in TB/HIV coinfection included a CD4 T cell count of < 50 cells/mm^3^, absence of antiretroviral therapy during TB treatment, and the presence of diabetes mellitus. In addition, individuals with TB/HIV coinfection had a higher occurrence of adverse events during treatment compared with HIV-negative individuals [37, 38].

In addition, a previous study highlighted that individuals with TB/HIV coinfection who were elderly (> 50 years of age) had a higher mortality rate compared with younger populations [38]. Elderly individuals with TB are challenging to treat due to nonspecific symptoms, atypical presentations, high lost-to-follow-up rates, and adverse drug events. The presence of comorbidities in older individuals with TB makes it difficult to accurately determine the cause of death. In addition, low body weight (< 45 kg) was associated with increased mortality during TB treatment, possibly due to impaired immunity and more severe infections in underweight individuals. Addressing these challenges requires a multidisciplinary approach, including social support, timely interventions, and tailored treatment strategies [37].

## 5. HIV Disease Progression

HIV disease progression significantly contributes to accelerated aging in older PLWH through mechanisms such as chronic immune activation, inflammation, and oxidative stress. Persistent immune activation leads to the release of proinflammatory cytokines and oxidative molecules that cause cellular damage, telomere shortening, and mitochondrial dysfunction.

### 5.1. Telomere Attrition

Telomeres, the protective caps at the ends of chromosomes, shorten with each cell division and are markers of cellular aging. In PLWH, telomere shortening occurs at an accelerated rate due to chronic inflammation, oxidative stress, and ongoing immune activation [39]. Studies show that individuals with perinatally acquired HIV exhibit significantly shorter telomeres compared with their HIV-negative peers, highlighting the long-term impact of the virus on cellular aging [40].

### 5.2. Mitochondrial Dysfunction

Mitochondrial dysfunction is another critical factor in the aging process for PLWH. Reduced mitochondrial DNA (mtDNA) content and increased production of reactive oxygen species (ROS) contribute to cellular damage, chronic inflammation, and the progression of age-related diseases [41]. ART exposure, while essential for viral suppression, can exacerbate mitochondrial dysfunction. Both treated and untreated PLWH exhibit lower mtDNA levels compared to healthy individuals, underscoring the need for interventions to mitigate this dysfunction [42–44].

### 5.3. Frailty

Frailty is a growing concern in older PLWH, characterized by diminished physiological reserve and increased vulnerability to stressors. Frailty complicates the management of HIV and associated comorbidities, increases the risk of hospitalization and disability, and heightens mortality rates. Addressing frailty requires comprehensive care that incorporates physical rehabilitation, nutritional support, and mental health services [45].

## 6. Polypharmacy and Antiretroviral Therapy

The introduction of ART has significantly improved the life expectancy of PLWH, transforming HIV from a fatal disease into a manageable chronic condition. However, long-term ART use is not without challenges, particularly for older PLWH. The cumulative effects of ART exposure, combined with the high prevalence of comorbidities in this population, contribute to polypharmacy and associated risks.

### 6.1. ART-Related Complications

ART has been instrumental in suppressing HIV replication and reducing immune activation, yet residual immune activation and chronic inflammation persist even in individuals on effective ART regimens [46]. Although ART effectively suppresses HIV replication and reduces immune activation, studies show that residual immune activation and inflammation can persist, contributing to chronic inflammation, immune dysregulation, and increased risks of non-AIDS-related comorbidities, such as cardiovascular and metabolic diseases. Proinflammatory markers, such as IL-6, CD8+ T-cell activation, and galectin-9, remain elevated, reflecting ongoing systemic inflammation. These inflammatory processes, established during acute HIV infection, often do not resolve fully with ART, leading to increased risks of non-AIDS-related comorbidities such as cardiovascular disease and metabolic disorders [47–50].

Certain ART drugs, particularly older nucleoside reverse transcriptase inhibitors (NRTIs) like stavudine and zidovudine, have been linked to mitochondrial toxicity, contributing to accelerated aging. These drugs impair mitochondrial function, increasing the production of ROS, which exacerbates oxidative stress and cellular damage. Protease inhibitors (PIs) further contribute to metabolic disturbances, including dyslipidaemia, insulin resistance, and lipodystrophy. These complications heighten the risk of cardiovascular diseases and other age-related conditions. Studies have shown that PIs can cause central fat accumulation, peripheral lipoatrophy, and increased carotid intima-media thickness (CIMT), a marker of atherosclerosis. In contrast, non-nucleoside reverse transcriptase inhibitors (NNRTIs) are associated with fewer metabolic disturbances but still present challenges for long-term management [51–54].

In addition, ART has been linked to epigenetic changes associated with aging. Some studies suggest that ART may reduce epigenetic age acceleration in HIV-infected individuals, potentially reversing some age-related changes. However, prolonged ART exposure is also associated with suboptimal CD4 recovery, weakened immune responses, and alterations in T-cell subsets, which can contribute to an aging phenotype characterized by diminished immune function [13].

### 6.2. Long-Term Implications of ART

Despite its benefits, ART contributes to accelerated aging through several mechanisms, including chronic immune activation, inflammation, and therapy-related side effects. Markers of monocyte activation, such as sCD14 and sCD163, remain elevated in PLWH on ART, indicating persistent inflammation and immune system activation. These markers are associated with increased risks of age-related diseases, including cardiovascular and neurocognitive disorders [55].

Furthermore, ART exposure has been linked to changes in body fat distribution, metabolic abnormalities, and neurocognitive impairments. These changes not only increase the risks of age-related diseases but also complicate adherence to treatment regimens, particularly in the presence of physical limitations and cognitive decline. Prolonged ART exposure is associated with slower CD4 recovery and faster CD4 decline when untreated, underscoring the need for tailored therapeutic strategies to address these challenges in older PLWH [56–58]. Addressing polypharmacy and optimizing ART regimens are critical to improving long-term outcomes for older PLWH. Strategies such as integrating multidisciplinary care teams, promoting adherence, and monitoring for drug interactions can mitigate the adverse effects of polypharmacy and enhance the quality of life for this population.

### 6.3. Polypharmacy in Older PLWH

Polypharmacy, defined as the concurrent use of multiple medications, is prevalent among older PLWH due to their increased burden of comorbidities. Studies indicate that approximately 82% of older PLWH take at least one non-HIV-related medication and 58% take five or more medications, placing them at higher risk for adverse drug reactions, drug–drug interactions, and nonadherence [10]. This threefold higher burden of comorbidities compared with HIV-negative counterparts complicates medication management and increases the risk of health complications [10]. Managing polypharmacy requires a holistic approach, including regular medication reviews, deprescription of unnecessary drugs, and optimization of ART regimens. Integrase inhibitor-based ART regimens, for example, have been associated with fewer drug–drug interactions, reduced systemic inflammation, and simplified treatment protocols. Switching to dual therapy has also shown promise in reducing medication burdens while maintaining viral suppression [18, 59].

## 7. Lifestyle and Socioeconomic Factors

Lifestyle factors can contribute to accelerated aging in individuals living with HIV. Smoking is more prevalent among PLWH compared with the general population. Smoking is more prevalent among PLWH than in the general population and exacerbates the risks of cardiovascular diseases, cancers, and other age-related conditions. The synergistic effects of smoking and HIV increase oxidative stress, inflammation, and immune dysfunction, accelerating the aging process [60]. Moreover, substance abuse, including alcohol and illicit drugs, is associated with increased oxidative stress, organ damage, and chronic inflammation. These factors compound the challenges faced by PLWH and contribute to premature aging. Poor dietary habits and malnutrition impair immune function and increase inflammation, further impacting health outcomes. Regular physical activity, on the other hand, has been shown to reduce inflammation, improve cardiovascular health, and enhance overall wellbeing in PLWH [61]. Mental health conditions, such as depression and anxiety, are more prevalent among PLWH [62]. Chronic stress and mental health disorders can contribute to accelerated aging through various mechanisms, including increased inflammation and oxidative stress [5].

## 8. Conclusions

The increasing population of older PLWH presents significant challenges for healthcare systems worldwide. With longer life expectancies driven by the success of ART, understanding the multifaceted factors contributing to aging with HIV is essential. These factors include immunosenescence, coinfections, disease progression, ART-related complications, polypharmacy, lifestyle influences, and socioeconomic determinants. Tailored, multidisciplinary interventions are crucial to address the unique needs of this demographic, improving health outcomes and quality of life in older PLWH as they navigate the complexities of aging with HIV.

## Data Availability

No new data were created or analyzed in this study. Data sharing is not applicable to this article.
